# Effect of malaria transmission reduction by insecticide-treated bed nets (ITNs) on the genetic diversity of *Plasmodium falciparum* merozoite surface protein (MSP-1) and circumsporozoite (CSP) in western Kenya

**DOI:** 10.1186/1475-2875-12-295

**Published:** 2013-08-27

**Authors:** Simon K Kariuki, James Njunge, Ann Muia, Geofrey Muluvi, Wangeci Gatei, Feiko ter Kuile, Dianne J Terlouw, William A Hawley, Penelope A Phillips-Howard, Bernard L Nahlen, Kim A Lindblade, Mary J Hamel, Laurence Slutsker, Ya Ping Shi

**Affiliations:** 1 Centre for Vector Biology and Control Research, Kenya Medical Research Institute, Kisumu, Kenya; 2 Biochemistry and Biotechnology Department, Kenyatta University, Nairobi, Kenya; 3 Atlanta Research and Education Foundation, Atlanta, GA, USA; 4 Centers for Disease Control and Prevention, Division of Parasitic Diseases and Malaria, Malaria Branch, Atlanta, GA, USA; 5 Child and Reproductive Health Group, Liverpool School of Tropical Medicine, Liverpool, UK; 6 President’s Malaria Initiative, Washington, DC, USA

**Keywords:** Malaria, Parasite diversity, MSP-1, CSP, Transmission, Bed nets

## Abstract

**Background:**

Although several studies have investigated the impact of reduced malaria transmission due to insecticide-treated bed nets (ITNs) on the patterns of morbidity and mortality, there is limited information on their effect on parasite diversity.

**Methods:**

Sequencing was used to investigate the effect of ITNs on polymorphisms in two genes encoding leading *Plasmodium falciparum* vaccine candidate antigens, the 19 kilodalton blood stage merozoite surface protein-1 (MSP-1_19kDa_) and the Th2R and Th3R T-cell epitopes of the pre-erythrocytic stage circumsporozoite protein (CSP) in a large community-based ITN trial site in western Kenya. The number and frequency of haplotypes as well as nucleotide and haplotype diversity were compared among parasites obtained from children <5 years old prior to the introduction of ITNs (1996) and after 5 years of high coverage ITN use (2001).

**Results:**

A total of 12 MSP-1_19kDa_ haplotypes were detected in 1996 and 2001. The Q-KSNG-L and E-KSNG-L haplotypes corresponding to the FVO and FUP strains of *P*. *falciparum* were the most prevalent (range 32–37%), with an overall haplotype diversity of > 0.7. No MSP-1_19kDa_ 3D7 sequence-types were detected in 1996 and the frequency was less than 4% in 2001. The CSP Th2R and Th3R domains were highly polymorphic with a total of 26 and 14 haplotypes, respectively detected in 1996 and 34 and 13 haplotypes in 2001, with an overall haplotype diversity of > 0.9 and 0.75 respectively. The frequency of the most predominant Th2R and Th3R haplotypes was 14 and 36%, respectively. The frequency of Th2R and Th3R haplotypes corresponding to the 3D7 parasite strain was less than 4% at both time points. There was no significant difference in nucleotide and haplotype diversity in parasite isolates collected at both time points.

**Conclusion:**

High diversity in these two genes has been maintained overtime despite marked reductions in malaria transmission due to ITNs use. The frequency of 3D7 sequence-types was very low in this area. These findings provide information that could be useful in the design of future malaria vaccines for deployment in endemic areas with high ITN coverage and in interpretation of efficacy data for malaria vaccines based on 3D7 parasite strains.

## Background

Malaria caused by *Plasmodium falciparum* continues to place a heavy burden on the health and economic development of populations in endemic areas [1]. Over the last decade, increased funding for malaria control has resulted in rapid scale-up of key interventions including insecticide-treated bed nets (ITNs) in many areas [2] ITNs have been shown to reduce malaria transmission by 70-90%, leading to significant reductions in human-vector contact, the number of infectious mosquito bites [3,4] and the force of infection [5]. Previous studies have shown that the intensity of malaria transmission is an important determinant of the malaria burden in the population, the rates of acquisition of protective immunity and the dynamics of parasite dispersal in mosquito vectors [6]. Based on these observations, fears have been expressed that reduced transmission and exposure to malaria parasites could modify the vector-host-parasite interaction, delay the acquisition of protective immunity and result in a shift in the burden of severe disease and mortality [7]. Studies designed to investigate whether sustained use of ITNs during infancy leads to an increase in malaria-related morbidity and mortality in older children demonstrated that there is no shift in the malaria burden [8,9]. The few studies that have assessed the impact of ITNs on acquired immunity by comparing antibody responses to malaria antigens in children with or without ITNs have been inconclusive [10-14].

However, there is limited information on the impact of transmission reduction as a result of sustained use of ITNs on the genetic diversity of *P*. *falciparum* parasites. Malaria transmission intensity has been shown to play an important role in determining the genetic diversity of malaria parasites [15]. This is because malaria parasites undergo an obligate sexual reproduction in the mosquito vector, thereby creating an opportunity for generation of parasite diversity through intragenic recombination and re-assortment of genetic material during meiosis [16]. In areas of high transmission, both the numbers of infected hosts and the carriage of multiple-clone infections per individual are generally higher, therefore increasing the probability for mosquito vectors to obtain genetically diverse gametocytes during blood feeding [16,17]. This in turn increases the chance of out-breeding and higher rates of genetic recombination. These observations have lead to the hypothesis that significant reduction in the intensity of malaria transmission could lead to a decrease in the mean number of multiple-genotype infections in humans, which could decrease the frequency of recombination resulting in a reduction in the level of genetic diversity [16].

A previous study conducted by this same group using neutral microsatellite (MS) markers has shown an overall stability in genetic diversity of *P*. *falciparum* after five years of high ITN coverage except for a significant MS locus specific change associated with gametocytes [18]. The current study was designed to investigate the impact of sustained use of ITNs on the genetic diversity of two leading *P*. *falciparum* vaccine candidate antigens during the same study period and in the same study area in western Kenya. This study assessed single nucleotide polymorphisms (SNPs) in genes encoding the relatively conserved B-cell epitopes of the blood-stage 19-kDa region of the merozoite surface protein (MSP-1 _19kDa_) and the highly polymorphic pre-erythrocytic-stage circumsporozoite protein (CSP) Th2R and Th3R T-helper cell epitopes. These two genes were chosen for assessment because their polymorphism is thought to be partly driven by the host immune pressure and are likely to be subjected to the diversifying effects of changes in malaria transmission [15]. Monitoring the genetic diversity of leading *P*. *falciparum* vaccine candidate antigens over time will help better understand the relationship between transmission intensity as malaria interventions are scaled up and the diversity of parasite antigens that are under selection pressure. In addition, these data will be useful in interpreting efficacy data of malaria vaccines that are based on 3D7 sequences.

The merozoite surface protein-1 (MSP-1) is the major protein on the surface of asexual blood stages of the malaria parasites [19]. The MSP-1 195-kDa protein is synthesized during schizont maturation and undergoes two rounds of proteolytic cleavage leaving a 19-kDa fragment (MSP-1 _19kDa_) adhering on the surface of merozoites during erythrocyte invasion [20]. The MSP-1 _19kDa_ contains two cysteine-rich epidermal growth factor (EGF)-like motifs that play an important role in erythrocyte invasion, making it an ideal target of inhibitory antibodies for blocking parasite invasion of erythrocytes [20] and therefore an ideal candidate for a malaria vaccine. Although MSP-1 is characterized by extensive polymorphisms, the 19-kDa region is relatively conserved apart from five key non-synonymous SNPs corresponding to amino-acid positions 1644 (Q/E), 1691 (K/T), 1699 (S/N), 1700 (N/S), and 1701 (G/R). Intragenic recombination and single-nucleotide mutations are thought to play a role in generation of allelic variation in MSP-1 _19kDa_[21,22]. Several malaria blood-stage vaccine candidates containing MSP-1 _19kDa_ have undergone field trials with varied results [23].

The CSP is the predominant protein found on the surface of sporozoites [24]. The *P*. *falciparum csp* gene encodes a protein of approximately 420 amino acid residues with a molecular weight of 58 kDa consisting of two non-repetitive regions at the 5' and 3' ends and a variable central region that encodes immunodominant B-cell epitopes with multiple repeats of four amino acid motifs [25]. The C-terminal end of the gene contains two highly polymorphic T-helper cell epitopes, Th2R and Th3R, flanking the conserved RII region [25]. The CSP has been the subject of extensive research for the development of a vaccine against pre-erythrocytic stages of malaria parasites [26]. The central repeat and C-terminal regions of the CSP forms part of the most clinically advanced malaria vaccine candidate RTS,S that is currently undergoing a phase III clinical trial in several African countries [27,28]. Studies conducted in areas of different transmission settings have shown an association between the genetic diversity in the *csp* gene and malaria transmission, with the highest diversity observed in parasite populations from Africa [15].

The current study investigated the impact of transmission reduction due to the use of ITNs on the genetic diversity of genes encoding two *P*. *falciparum* polymorphic vaccine candidate antigens in 1996 before and 5 years after the deployment of ITNs in an area of western Kenya holoendemic for malaria. Prior to the ITN trial, malaria transmission in this area was intense and perennial with an estimated entomologic inoculation rate (EIR) of 60 to 300 infective bites per person per year [29]. It was estimated that malaria transmission in this area reduced by 95% after the introduction of ITNs [9].

## Methods

### Study area and population

This study was conducted in the context of a community-based cluster randomized controlled trial of ITNs in western Kenya that consisted of two phases. Phase 1 was designed to assess the impact of ITNs on all-cause mortality in young children while phase 2 consisted of an extended surveillance to monitor whether sustained use of ITNs during infancy increased all-cause morbidity and mortality in older children. The characteristics of the study area, design of the ITN trial and the two-year surveillance have been described in detail elsewhere [9,30]. Briefly, a randomized ITN trial was conducted between January 1997 and March 1999 in Rarieda Division (Asembo) where malaria is holoendemic and transmission occurs throughout the year.

At the end of the two-year trial period, ITNs were also distributed to all control villages and monitoring continued for an additional two years until March 2001. Throughout the two phases, annual cross-sectional surveys were conducted in 60 villages to collect information on malaria morbidity indicators. During the surveys finger stick blood samples were taken among children aged below five years. In addition, entomologic indices were collected throughout the two phases of the trial to estimate malaria transmission. During phase 1 of the trial there was a 72% reduction in the density of indoor-resting blood-fed *Anopheles* in the houses with ITNs and in the second phase, villages with ITNs experienced a 77% reduction in Anopheles densities compared with villages in an adjacent area [9]. In addition, parasite prevalence in children under the age of five decreased from 70% prior to the ITN trial to 34% in 2001 [9,31]. The use of ITNs before the trial was less than 5% but increased to 65.9% and 82.5% in phase 1 and 2 respectively [9].

The blood samples used in the current study were collected in October and November 1996, before phase 1 of the ITN trial and in May and June in 2001, 5 years after introduction of ITNs in phase 1. Microscopically confirmed malaria positive blood samples were randomly selected from those collected in the 1996 cross-sectional survey and matched by village with samples collected in the 2001 survey.

### Laboratory procedures

#### Sample collection

During each survey, 250-500 μl of blood was collected from each child by finger-stick into EDTA microtainers (Becton Dickinson, Franklin Lakes, NJ). Blood samples were transported in cool boxes to the central laboratory in Kisian, about 50 km from the field site and stored at 4°C until further processing. All samples were labelled with unique identification numbers that could not be linked to the randomization status of the sampled children. Erythrocytes were separated from plasma by centrifugation at 700 G for 5 min. Packed red blood cells (RBCs) were aliquoted into sterile vials and stored at ^-^70°C until the day of DNA isolation.

#### DNA extraction and PCR amplification

Parasite genomic DNA was extracted using the QIAamp® DNA Mini Kit (Qiagen, Hilden, Germany) according to the manufacturer’s instructions. The CSP gene was amplified by polymerase chain reaction (PCR) in a 25 μl reaction mixture containing 1 μl of DNA, 0.2 μM of forward and reverse primers CSP-F: ACAATCAAGG TAATGGACAAGG and CSP-R:GGATTAATAATGGTATTATCCTTCT, 1× TBE buffer, 2.0 mM MgCl_2_, 250 μM dNTPs, and 2.5 U Taq DNA polymerase (Promega Corporation, Madison, Wisconsin, USA). The cycling conditions were as follows: 95°C for 5 min, 58°C for 2 min and 72°C for 2 min for 1 cycle, then 94°C for 1 min, 58°C for 2 min and 72°C for 1 min for 32 cycles followed by 10 min extension at 72°C. The primers were designed to hybridize to conserved regions covering the Th2R, Th3R and the universal T-cell epitopes, resulting in a 354 base-pair fragment. The MSP-1_19-kDa_ gene was amplified in a 25 μl reaction mixture containing 1 μl of DNA, 0.2 μM of two forward primers- 5’ CGTTTTATCTAATTTACTTGATGGAA 3’(K16F), specific for K1 and 5’ CCTAATACAATAATATCAAAATTAATTGA 3’ (M16F), specific for MAD-20 and a common reverse primer 5’ TTAAGGTAACATATTTTAACTCCTAC 3’ (C3flaR), 2.0 mM MgCl_2_, 200 μM dNTPs, and 1.25U Taq DNA polymerase with the following cycling conditions: 91°C for 1.5 min, then 91°C for 30 sec, 50°C for 40 sec and 70°C for 40 sec for 40 cycles followed by 5 min extension at 72°C. PCR was carried out in a GeneAmp® PCR System 9700 (Biosystems, PE, USA).

#### Sequencing

The PCRs products were cleaned using CentriSep spin columns (Princeton Separations, Adelphia, NJ) according to the manufacturer instructions. Sequencing reactions were carried out using forward and reverse primers in separate reactions by the BigDye® Terminator v3.1 Cycle Sequencing Kit and analysed in an ABI 3100 DNA Sequencer (Applied Biosystems, Foster City, CA). The ChromasPro, version 2.31 software (Technelysium Pty Ltd, Queensland, AU) was used to align the sequences. Each sequence was manually checked for true base calling and samples with doubtful sequencing results were repeated.

### Data analysis

The MSP-1_19-kDa_ sequences were aligned with known sequences from the *P*. *falciparum* genome database at the National Center for Biotechnology Information (NCBI) using the *P*. *falciparum* clone 3D7 as the reference sequence while the CSP Th2R and Th3R sequences were aligned using the 3D7 and 7G8 clones. Molecular analyses were conducted using MEGA version 4 [32] and DNA Sequence Polymorphism, version 4.0 [33] softwares to create haplotypes. Differences in the frequency of haplotypes between baseline and post-ITN intervention surveys were compared by Chi-square test. The genetic diversity parameters including nucleotide diversity (π), number of haplotypes (NHap) and haplotype diversity (HapDiv) in parasite populations at baseline and at post ITN intervention were determined using the sequence polymorphism softwares, DnaSP v5 [34], MEGA version 4 [32] and ARLEQUINS suite ver 3.5 [35]. Nucleotide sequences reported here are available in GeneBank under accession numbers BankIt1630040: KF158421 - KF158555 (*Pfmsp*-*1*_*19*-*kDa*_) and BankIt1630320: KF158556 - KF158712 (*Pfcsp* C-terminal domain).

### Ethical approval

The study was approved by the Ethical Review Committee of the Kenya Medical Research Institute, Nairobi, Kenya and the Institutional Review Board of the Centers for Disease Control and Prevention (CDC) Atlanta, Georgia, USA.

### Consent

Written informed consent was obtained from the patient’s guardian/parent/next of kin for the publication of this report and any accompanying images.

## Results

### Frequency of MSP-1_19-kDa_ haplotypes

**Figure 1:**
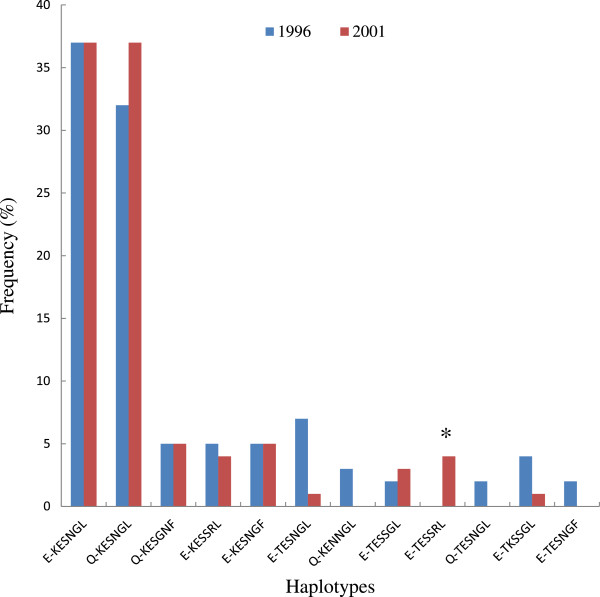
**The frequency of *MSP*-*1***_***19*-*kDa***_**haplotypes at baseline (1996) and following 5 years of community-wide ITN use (2001).** * Indicates the haplotype sequence corresponding to the 3D7 *P*. *falciparum* strain.

A total of 136 parasite samples were successfully sequenced for the gene encoding MSP-1_19-kDa_, 58 at baseline and 78 in 2001. Overall, seven non-synonymous amino acid replacements were detected at positions 1644 (Q-E), 1691 (K-T), 1695 (E-K), 1699 (S-N), 1700 (S-N), 1701 (G-R) and 1716 (L-F). Based on the amino acid replacements, a total of 12 MSP-1_19-kDa_ haplotypes were detected in the study area in 1996 and 2001. Out of these, 3 haplotypes, Q-TESNGL, E-TESSGL and E-TESNGF are new and have not been reported before. Out of the 12 haplotypes identified, 8 haplotypes were observed in the 1996 and 2001 surveys while 4 haplotypes were observed at only one time point, 2 in 1996 and 2 in 2001. The E-KSNGL and Q-KSNGL haplotypes corresponding to the FUP-Uganda PA and FVO-Wellcome strains of *P*. *falciparum* had the highest prevalence in 1996, 37 and 32% respectively, and in 2001 it was, 37% for both strains (Figure 1). The frequency of all the other haplotypes was low, ranging from 2-7% at both time points. The frequency of the MSP-1 haplotype (E-TESSRL) corresponding to the 3D7 strain, the sequence contained in malaria vaccine candidates that are at various stages of development was less than 4% in 2001 and none was detected in 1996.

### Genetic diversity of the MSP-1_19-kDa_ haplotypes

The genetic diversity of the MSP-1_19-kDa_ in parasite populations in 1996 and 2001 were comparable, with a nucleotide diversity (π) of 0.0038 and 0.0033 respectively (Table 1). Likewise, there was no significant difference in the number of haplotypes (NHap) or haplotype diversity (HapDiv) in parasite isolates collected in 1996 and 2001.

**Table 1:** **Genetic diversity at the MSP-1**_**19-kDa **_**domain at baseline (1996) and following 5 years of community-wide ITN use (2001)**

**MSP-1**_**19-kDa **_**domain**				
**Survey**	**n**	**π**	**NHap**	**HapDiv**
1996	58	0.0038 (0.0005)	11	0.782
2001	78	0.0033 (0.0004)	9	0.721

n, Number of samples sequenced per cross-sectional survey.

π, Nucleotide diversity (standard deviation).

Nhap, No of haplotypes.

HapDiv, Haplotype diversity.

### Frequency of CSP Th2R and Th3R haplotypes

**Figure 2:**
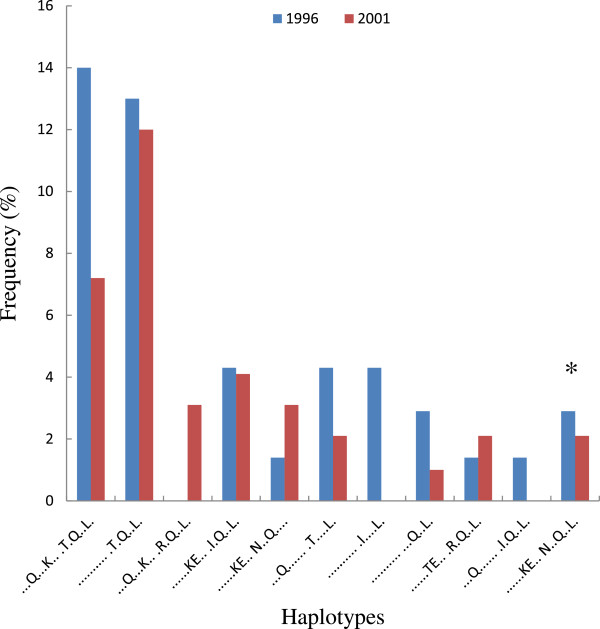
**The frequency of most common Th2R CSP haplotypes at baseline (1996) and following 5 years of community-wide ITN use (2001).** * Indicates the haplotype sequence corresponding to the 3D7 *P*. *falciparum* strain.

**Figure 3:**
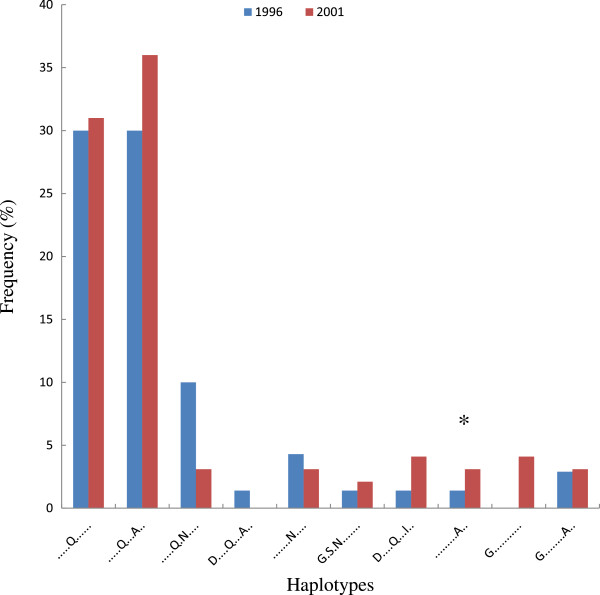
**The frequency of the most common Th3R CSP haplotypes at baseline (1996) and following 5 years of community-wide ITN use (2001).** * Indicates the haplotype sequence corresponding to the 3D7 *P*. *falciparum* strain.

At both surveys, a total of 157 (68 in 1996 and 89 in 2001) out of 175 parasite samples yielded interpretable sequences and were included in the analysis. Overall, there was a high diversity on the CSP C-terminal domain containing immunodominant T-cell Th2R and Th3R epitopes (Figures 2 and Figure 3). At the Th2R domain, a total of 26 and 34 haplotypes were detected in 1996 and 2001 surveys respectively, and for the Th3R domain, these were 14 and 13 haplotypes. Overall, there was a high fluctuation in the frequency of both the Th2R and Th3R haplotypes at both time points with some haplotypes detected at baseline and not in 2001 and vice versa (Figures 3A and B). Two Th2R haplotypes, QHIEKYLKTIQNSL and QHIEQYLKTIQNSL, had the highest frequency in 1996; 14 and 13% respectively; and 2001, 7 and 12% respectively. The frequency of all the other Th2R haplotypes was less than 5% at both time points. At the Th3R domain, the NKPKDEQDYEND and NKPKDEQDYAND haplotypes had the highest frequencies, in 1996, 30% for both haplotypes; and in 2001, 31 and 36% respectively. The frequency of all the other Th3R haplotypes was less than 10%. At both Th2R and Th3R domains, the frequencies of haplotypes corresponding to the 3D7 strain of *P*. *falciparum* were less than 4% at both time points.

### Genetic diversity of the CSP Th2R and Th3R haplotypes

The genetic diversity of the CSP gene at the Th2R and Th3R domains are shown in Tables 2 and Table 3. Similar to the high number of haplotypes detected in 1996 and 2001, there was a high diversity in both domains at the two time points. The genetic diversity was higher in Th2R than the Th3R region as seen in the higher haplotype and nucleotide diversity (HapDiv and π) at both time points. There was no significant difference in the genetic diversity at both T cell epitopes between parasite isolates collected in the two surveys.

**Table 2:** Genetic diversity at the CSP Th2R epitope at baseline (1996) and following 5 years of community-wide ITN use (2001)

**CSP Th2R**				
**Survey**	**n**	**π**	**NHap**	**HapDiv**
1996	68	0.0594 (0.00039)	26	0.943
2001	89	0.0613 (0.00035)	34	0.956

n, Number of samples sequenced per cross-sectional survey.

π, Nucleotide diversity (standard deviation).

Nhap, No of haplotypes.

HapDiv, Haplotype diversity.

**Table 3:** Genetic diversity at the CSP Th3R epitope at baseline (1996) and following five years of community-wide ITN use (2001)

**Th3R**				
**Survey**	**n**	**π**	**NHap**	**HapDiv**
1996	68	0.0549 (0.0068)	14	0.811
2001	89	0.0437 (0.0052)	13	0.779

n, Number of samples sequenced per cross-sectional survey.

π, Nucleotide diversity (standard deviation).

Nhap, No of haplotypes.

HapDiv, Haplotype diversity.

## Discussion

For the gene encoding the blood stage MSP-1_19-kDa_ antigen, a total of 12 haplotypes were identified at both baseline and 5 years after distribution of ITNs in this area. This number is consistent with findings from previous studies conducted in our study area and in other malaria holoendemic areas of Africa showing a higher number of MSP-1_19-kDa_ haplotypes (range 6-15) in areas of high transmission and high EIRs ranging from 50 to 600 infective bites person per year, [21,22,36] compared with fewer haplotypes (range 1-3) in areas of lower transmission and EIRs of less than 10 infective bites per person per year [37,38]. The E-KSNGL haplotype corre-sponding to the FUP-Uganda PA parasite strain and the Q-KSNGL corresponding to the FVO-Wellcome strain were the most predominant MSP-1_19-kDa_ haplotypes in 1996 and 2001. These two haplotypes have been reported at high frequencies in previous studies conducted in this area and other regions in Africa. In a study conducted in the same area in 1998, Qari *et al* reported frequencies of 42 and 21% for the E-KSNGL and Q-KSNGL haplotypes respectively [21]. Similarly, frequencies of 36 and 38% for the E-KSNGL and Q-KSNGL haplotypes respectively have been reported in a site near this study area [39]. In the current study, the frequency of other MSP-1_19-kDa_ haplotypes was very low.

There was no significant change overtime in the frequency, the number of MSP-1_19-kDa_ haplotypes, haplotype diversity and π. The stability in the frequency of haplotypes and the genetic diversity overtime, suggests that the *MSP*-*1*_*19*-*kDa*_ gene could be under balancing selection as documented in previous studies [40-42]. Similar levels of genetic stability have been reported in a malaria holoendemic area of Tanzania where polymorphic sites of the gene encoding the *MSP*-*1*_*19*-*kDa*_ gene was relatively stable for a period of 15 years [22]. This contrasts with significant temporal variations in the frequencies of MSP-1_19-kDa_ haplotypes reported in a study conducted in an area of low malaria transmission [43]. This difference has been explained to be due to the relatively low rates of meiotic recombination or strong selection pressure that maintain a clonal structure in parasite populations from areas of low transmission, while the reverse is true in areas of high transmission [43]. Therefore, the relative stability of *MSP*-*1*_*19*-*kDa*_ haplotypes observed in this study over a period of 5 years suggests that the rates of meiotic recombination in parasite populations in this area remain high despite the significant reduction in transmission associated with the deployment of ITNs.

The frequency of the MSP-1 haplotype (E-TESSRL) corresponding to the 3D7 strain of *P*. *falciparum* was not detected at baseline and was less than 4% in 2001. Low frequencies of MSP-1 3D7 sequence-types has been reported in previous studies conducted in different malaria transmission areas [21,36,43,44]. It has been suggested that the low frequency of haplotypes corresponding to the 3D7 strain of *P*. *falciparum* could compromise the efficacy of MSP-1 vaccines that are based on the 3D7 clone. For example, a recent phase IIb trial of an MSP-1_42_-based vaccine candidate derived from the 3D7 clone of *P*. *falciparum* in children aged 12-27 months in a nearby study area reported an overall vaccine efficacy of 5.1% [45]. The poor efficacy was partly attributed to the high multiplicity of infections and diversity of MSP-1_42_ gene in this area [45]. Similarly, the variable success of MSP-1 vaccines that are at various stages of development including those based on FVO alleles has been attributed to polymorphisms in *P*. *falciparum* antigens [46]. Therefore, data on the genetic diversity of parasite populations circulating in area similar to that obtained in the current study provide important baseline information that could be useful in planning and interpreting data on the efficacy of vaccines that are based on polymorphic antigens such as MSP-1.

For the highly polymorphic Th2R and Th3R T-helper cell epitopes, there were 26 and 34 Th2R haplotypes at baseline and 5 years post-ITNs respectively and 14 and 13 Th3R haplotypes at the two time points. These findings are consistent with results of an earlier study conducted in the same area that reported 29 and 16 haplotypes for the Th2R and Th3R epitopes respectively [47]. This suggests that the CSP haplotypes circulating in this area have remained relatively stable for an extended period. This contrasts sharply with findings from areas of low malaria transmission which show that 1 or 2 haplotypes are predominant with frequencies as high as 80% [48]. In this study, the most frequent Th2R and Th3R haplotypes were 14% and 30% at baseline and 13 and 36% in 2001. Other haplotypes were rare with a prevalence of less than 10%. The frequency of haplotypes corresponding to the 3D7 parasite strain was less than 4%. The RTS,S malaria vaccine candidate that is currently undergoing a phase 3 efficacy trial in areas of different transmission intensities in Africa [27,28] is derived from the 3D7 strain of *P*. *falciparum* and comprises part of the repeat, Th2R and Th3R regions of the CSP. Although there is no evidence that the observed efficacy of the RTS,S vaccine is sequence-dependent [49,50] the information obtained in this study could be useful in the interpretation efficacy data of the RTS,S vaccine and other CSP-based malaria vaccines that are based on the 3D7 parasite strain in this area. This information could also inform the design of future vaccines for deployment in areas of high and perennial malaria transmission.

The high genetic diversity in the T-cell epitopes of the *CSP* gene was also apparent when measured by haplotype diversity and π. These findings are consistent with previous studies that have documented high genetic diversity in ThR2 and Th3R regions of CSP in parasite populations from regions of high malaria transmission [47,51,52]. This contrasts with low genetic diversity in parasite populations from areas of low malaria transmission of Brazil [52], Papua New Guinea [48,53], India [54] and Iran. In addition, similar to previous studies, the current study also shows that the Th2R region is more diverse than the Th3R region, suggesting that the Th2R region could be under strong selective pressure unrelated to the intensity of malaria transmission. Similar to *MSP*-*1*_*19*-*kDa*_ gene, there was no significant difference in genetic diversity of the two T-cell epitopes in parasite populations at baseline and 5 years post-ITN intervention. The relative stability in the diversity of Th2R and Th3R regions has also been documented over a period of 5 years in a region of low transmission in Vietnam [55]. Taken together, our results indicate that transmission reduction due to sustained use of ITNs over a period of five years had no impact on the genetic diversity of the highly polymorphic Th2R and Th3R epitopes of the *CSP* gene.

Several factors have been suggested to play a role in the generation and maintenance of parasite diversity including transmission intensity, immune-mediated pressure, parasite positive rates, rates of multiple-genotype infections and patterns of antimalarial drug use [22]. In the current study, the sustained use of ITNs over a period of five years in a region of western Kenya with intense and perennial malaria transmission had no significant impact on parasite diversity of the two polymorphic *P*. *falciparum* genes. A few factors could explain this observation. Firstly, despite a decline in transmission intensity following the deployment of ITNs, parasite prevalence remained relatively high and 50% of children sleeping under ITNs were parasitaemic during phase 1 of the ITN trial (1996-1998) [31], and > 30% of post neonatal infants (one to 11 months) were parasitaemic during phase 2 of the trial (1999-2001) [9]. Similarly, cross-sectional surveys conducted 2-4 years after introduction of ITNs showed that up to one third of infants sleeping under ITNs were parasitaemic [9]. Secondly, although the target of the ITN trial was to cover all the sleeping spaces and efforts were made to monitor adherence, a significant proportion of people did not sleep under ITNs and remained exposed to malaria [56]. In phase 1 of the trial which distributed ITNs only to half of the villages, adherence to the use of ITNs was 65.9% and increased to 82.5% during the second phase when ITNs were distributed to the villages that acted as control in phase 1 [9]. Collectively, these epidemiological findings and the results obtained in the current study on stability of parasite genetic diversity suggest that exposure to malaria parasites in western Kenya remains at a level that allows genetic mixing of diverse parasite genes despite transmission reduction due to ITNs. Furthermore, it is possible that any observable impact of vector control interventions on genetic diversity of malaria parasites might require reduction of transmission intensity by more than the 90% reported in the ITN trial [4]. It is also conceivable that in areas of intense transmission, the impact of transmission reduction on parasite diversity will take greater reduction in transmission and for a much longer period than the 5-year period used to select sample for analysis in the current study or 15 years as shown in a previous study in Tanzania [22]. The short time period used in comparing parasite diversity after introduction of ITNs is a limitation in this study. The antigens used in this study could also be a limitation. Although the antigens used have been shown in previous studies to be under strong immunological pressure, it is likely that detection of changes in parasite diversity as a result of transmission reduction requires antigens that are highly sensitive to transmission dynamics. Lastly, although the study compared parasite diversity before and after introduction of ITNs, the absence of a contemporaneous area without ITNs for comparison is also a limitation in the current study.

## Conclusions

Data obtained in this study show that the MSP-1_19kDa_ haplotypes have remained stable overtime and transmission reduction due to ITNs had no impact on the diversity of MSP-1_19kDa_ and the Th2R and Th3R domains of the CSP. The results also show that the CSP Th2R region is more diverse than the Th3R region and could be under strong selective pressure unrelated to the intensity of malaria transmission. The information on low frequency of 3D7 clone could be useful in interpreting data on the efficacy of malaria vaccines based on the 3D7 parasite strain in areas of stable malaria transmission and in informing the development of future malaria vaccines for deployment in areas of high transmission.

## Competing interests

The authors declare they have no competing interests.

## Authors’ contributions

SK, YPS, FK, DT, WH, PP-H, BL KL, MH, and LS designed the study. SK, JN, AM, and GM collected the molecular biology data. JW, SK, YPS, WG, JN, AM participated in the data analysis. SK and YPS wrote the paper. All authors read and approved the final manuscript.
